# Vaccination Against Androgen Receptor Splice Variants to Immunologically Target Prostate Cancer

**DOI:** 10.3390/vaccines12111273

**Published:** 2024-11-13

**Authors:** Robert D. Marek, Selena Halabi, Mu-En Wang, Jason McBane, Junping Wei, Tao Wang, Xiao Yang, Congxiao Liu, Gangjun Lei, Herbert Kim Lyerly, Ming Chen, Timothy N. Trotter, Zachary C. Hartman

**Affiliations:** 1 Department of Pathology, Duke University, Durham, NC 27710, USA; rd.marek@duke.edu (R.D.M.); muen.wang@duke.edu (M.-E.W.);; 2 Department of Biomedical Engineering, Duke University, Durham, NC 27708, USA; 3 Duke Cancer Institute, Duke University, Durham, NC 2771, USA; kim.lyerly@duke.edu; 4 Department of Surgery, Duke University, Durham, NC 27777, USA; jason.mcbane@duke.edu (J.M.); junping.wei@duke.edu (J.W.); timothy.trotter@duke.edu (T.N.T.); 5 Department of Immunobiology, Duke University, Durham, NC 27708, USA

**Keywords:** prostate cancer, androgen receptor, vaccines, castration resistance, immune suppression, immune checkpoint inhibition

## Abstract

**Background/Objectives**: Androgen receptor (AR) expression and signaling are critical for the progression of prostate cancer and have been the therapeutic focus of prostate cancer for over 50 years. While a variety of agents have been developed to target this axis, many of these fail due to the emergent expression of AR RNA splice variants, such as AR-V7, that can signal independently of ligand binding. Other therapies, such as vaccination against prostate-specific antigens, have achieved FDA approvals but have fallen short of being incorporated as standard-of-care therapies for advanced prostate cancer. This may be due to the elevated level of immunosuppression observed in prostate cancer, which remains largely refractory to immune checkpoint blockade. **Methods**: We developed a vaccine targeting AR-V7, a common isoform associated with treatment resistance, and demonstrated its ability to elicit AR-V7-specific immunity and enable anti-tumor responses against AR-V7+ cancers in subcutaneous tumor models. **Results**: Our studies also revealed that AR-V7 expression conferred an immune suppressive phenotype that was significant in a non-AR-dependent prostate cancer model. Notably, in this model, we found that vaccination in combination with enzalutamide, an AR antagonist, suppressed these aggressive immune suppressive cancers and resulted in enhanced survival in comparison to control vaccinated and enzalutamide-treated mice. While anti-PD-1 immune checkpoint inhibition (ICI) alone slowed tumor growth, the majority of vaccinated mice that received anti-PD-1 therapy showed complete tumor elimination. **Conclusions**: Collectively, these results validate the importance of AR signaling in prostate cancer immune suppression and suggest the potential of AR-V7-specific vaccines as therapeutic strategies against prostate cancer, offering significant protective and therapeutic anti-tumor responses, even in the presence of androgen signaling inhibitors.

## 1. Introduction

Prostate cancer (PC) is one of the most prevalent cancers among men and remains the second leading cause of cancer-related death in men. For decades, effective prostate cancer treatments have disrupted the androgen receptor (AR) signaling pathway through androgen deprivation therapies (ADTs). As ~90% of PCs are dependent on AR signaling at diagnosis [1], ADT initially inhibits cancer cell proliferation by lowering circulating testosterone concentrations, but ~10–20% of cases will progress and develop castration-resistant prostate cancer (CRPC) within 5 years [2]. Next-generation antiandrogens, enzalutamide (AR antagonist) and abiraterone (inhibitor of cytochrome P17A1, which is required for androgen biosynthesis), have shown some clinical benefits in CRPC, but progression remains inevitable in metastatic cases [3]. Multiple resistance mechanisms have been implicated in the development of castration-resistant prostate cancer (CRPC), including local production of testosterone, overexpression of AR and the expression of constitutively active AR RNA splice variants (AR-Vs) [1,4,5].

Constitutively active AR-Vs lack a functional ligand-binding domain (LBD), but retain the N-terminal domain (NTD), DNA-binding domain (DBD), and hinge region [6]. While AR is traditionally dependent on ligand-binding for nuclear localization, constitutively active AR-Vs are imported to the nucleus in the absence of ligands and have transcription factor activity [7]. Among the different AR-Vs, androgen receptor RNA splice variant 7 (AR-V7) is the most widely studied and highly expressed variant, associated with resistance to multiple next-generation antiandrogens [4,8]. Resistance to AR-targeted therapies has been associated with AR-V7 expression in large clinical data sets, resulting in accelerated progression and poorer survival outcomes [1,4,9]. Multiple studies have shown AR-V7 to be capable of transcription factor activity independent of ligand binding, which can activate signaling pathways distinct from that of ligand-dependent AR [10,11,12]. The AR-V7 transcript contains the first three exons of AR and terminates with splicing to a cryptic exon, encoding a unique 16 amino acid sequence at the truncated c-terminus of the protein [13]. This truncated form of AR is undetectable at the protein level in normal benign tissues, but is found in up to 75% of metastatic cases [14]. This clinically functional importance and unique cancer specificity makes AR-V7 a potentially promising target for immunotherapeutic vaccination.

The key to the success of any cancer vaccination strategy is the selection of an antigenic target that results in an immune response that can overcome self-tolerance and the immunosuppressive nature of tumor microenvironments. In our previous studies using HER2-specific vaccines, an oncogenic driver for HER2+ breast cancer, we found that targeting this gene and signaling axis conferred significant anti-tumor efficacy and inhibition of tumor growth, while vaccination against a non-oncogenic tumor-associated antigen (EGFP), showed no effect on tumor growth despite potent antigen-specific immunity against EGFP [15]. Given these findings, we hypothesized that vaccines targeting constitutively active AR-Vs could be more efficacious than vaccines targeting tumor-associated antigens like PSA or PAP. Supportive of this approach, we have found that in preclinical ER+ breast cancer models, vaccination against estrogen receptor alpha (ESR1) gain of function mutants results in anti-tumor responses against ESR1-mutant-expressing murine mammary cancer models [16].

To test this hypothesis, we developed an adenoviral vaccine against AR-V7 (Ad-AR-V7) and demonstrated its ability to elicit antigen-specific T cell responses against a variety of AR-V7 epitopes, predominantly to those in the N-terminus. We then expressed AR-V7 in an immunocompetent murine colorectal line known to respond to antigen-specific vaccination and demonstrated that adenoviral AR-V7 vaccination suppressed tumor growth and improved survival in both preventative and therapeutic vaccination strategies. When we expressed AR-V7 in a novel AR-negative murine prostate cancer cell line, we found evidence for androgen signaling and transcriptional stimulation of extracellular matrix production and MAPK/ERK signaling, as well as suppression of chemokine gene signatures. This resulted in an in vivo immunosuppressive benefit, mediated by CD8+ T cells. Finally, we found that Ad-AR-V7 alone had no significant benefit in this model but could suppress tumor growth and improve survival in combination with enzalutamide or PD-1 checkpoint blockade.

## 2. Materials and Methods

### 2.1. Cell Culture and Viral Vector Construction

Cell lines (HEK293T, CT26, and P3CA) were maintained in DMEM and 10% heat-inactivated fetal bovine serum. Cells were modified by stable lentivirus transduction. Lentiviral particles were added to medium containing 8 µg/mL polybrene, and cells were incubated for 48 h before selection was performed with hygromycin. Lentiviral and adenoviral constructs were generated by Gateway LR cloning (Invitrogen, Waltham, MA, USA) of entry plasmids into 3rd-generation lentiviral destination plasmids previously established in our lab or into a pAd-CMV-V5-DEST Gateway vector (Invitrogen). Entry plasmids containing AR, AR-V7, AR-V12, a control antigen, or an empty cassette without an ORF were generated using Geneblocks (Integrated DNA Technologies, Inc., Coralville, IA, USA) and Gibson Isothermal Assembly reactions (New England Biolabs, Ipswich, MA, USA). Lentivirus particles were generated in HEK293T cells by cotransfection with packaging and envelope plasmids followed by ultracentrifugation. Adenoviral particles were generated in 293 cell lines using standard techniques and were purified by CsCl gradient or AdEasy Virus Purification (Agilent, Santa Clara, CA, USA).

### 2.2. Luciferase Reporter Assay

Using PEI, HEK 293T cells were transfected with plasmid (0.2 µg DNA per 2 × 10^4^ cells) containing a transcription factor binding site to drive the expression of firefly luciferase. Plasmids specific to 45 different transcription factors were used. Cells were cultured in media without phenol red prepared with charcoal stripped HIFBS, and testosterone was added at 100 ng/mL. After 24 h, cells were lysed, and luciferase activity was measured on a Veritas Microplate Luminometer (Turner Biosystems, Sunnyvale, CA, USA).

### 2.3. mRNA Sequencing and Data Analysis

RNA from P3CA cell lines was extracted using an RNeasy column kit following the manufacturer’s protocol (QIAGEN, Venlo, The Netherlands). RNA samples were submitted to NovoGene (Novogene Corporation Inc., Sacramento, CA, USA) for cDNA synthesis, sequencing, and analysis. The top 30 differentially expressed genes are presented along with the most significantly enriched pathways found by GO pathway analysis.

### 2.4. Cell Line Flow Cytometry

P3CA cell lines were stained with an unconjugated rat anti-mouse H-2 antibody (M1/42, BioLegend, San Diego, CA, USA), and a BV421 goat anti-Rat IgG secondary (Poly4054, BioLegend). Cells were collected on a 3-Laser Northern Lights (Cytek Biosciences, Fremont, CA, USA) and analyzed with FlowJo v10.

### 2.5. Western Blot

Western blots were performed using a 4–15% gradient gel (BioRad, Hercules, CA, USA), and stained with anti-AR primary antibody (D6F11, Cell Signaling Technology, Danvers, MA, USA), followed by an IRDye 800CW secondary (LI-COR Biosciences, Lincoln, NE, USA). The blot was scanned on LI-COROdyssey CLx, stripped with Restore PLUS buffer (Thermo Scientific, Waltham, MA, USA), reprobed for β-actin (AC-15, Santa Cruz Biotechnology, Dallas, TX, USA) and IRDye 680RD secondary (LI-COR Biosciences), and was scanned again on the Odyssey CLx.

### 2.6. In Vivo Studies

SCID-beige (C.B-Igh-1b/GbmsTac-Prkdcscid-Lystbg N7; model CBSCBG) and BALB/c (BALB/cAnNTac; model BALB) animals were purchased from Taconic Biosciences (Germantown, NY, USA) and bred at Duke University. C57BL/6J (C57BL/6J, strain 000664) animals were purchased from The Jackson Laboratory (Bar Harbor, ME, USA). B6 albino mice (B6(Cg)-Tyrc-2J/J, strain 000058) were purchased from The Jackson Laboratory and bred at Duke University. Mice were used between 6 and 12 weeks of age. CT26 and P3CA cells were injected subcutaneously in the right flank of male mice anesthetized with isoflurane (2 × 10^5^ cells in 0.05 mL of DPBS per animal). Tumors were monitored by caliper measurements, and volumes were calculated using the following formula: volume = (length × width^2^) ÷ 2, where length is the larger of the two measurements. Survival was defined as tumors < 1000 mm^3^ or humane endpoint. Mice were vaccinated in the left quadriceps while anesthetized with isoflurane (1−2.6 × 10^10^ particles in 0.05 mL per mouse). Mice treated with enzalutamide were given an enzalutamide diet (50 mg/kg in 5053, Envigo, resulting in approximately 0.25 mg per mouse per day) [17]. CD8 depleting antibodies were given IP, (200 µg, followed by 100 µg weekly doses). All animal studies were performed in accordance with Duke IACUC approved protocol (A080-20-04 and A043-23-02) and housed by the Division of Laboratory Animal Resources.

### 2.7. ELISpot Assays and Intracellular Cytokine Staining Flow Cytometry

Mouse splenocytes were collected by passing whole spleens through a 70 µm filter, and red blood cells were lysed with ACK buffer. IFNγ ELISPOT assays were performed following the manufacturer’s instructions (Mabtech Inc., Cincinnati, OH, USA). In summary, splenocytes (500,000 cells/well) were stimulated for 18 h with the indicated peptides at 1 µg/mL in RPMI with 10% HIFBS. Peptide pools contained 1 µg/mL of each peptide. PMA and ionomycin were used as positive controls. AR peptide pools were made using overlapping 15-mers spanning the length of AR with 4 amino acid overlaps ordered from GenScript. Peptides were dissolved in DMSO, and pooled stocks were made at 100 µg/mL/peptide. For a complete list of peptide sequences, see Supplementary Table S1. Flow cytometry was performed by stimulating splenocytes in the presence of brefeldin a and monensin, followed by surface and intracellular staining using a Foxp3 Transcription Factor Staining Kit (Cytek Biosciences). Cells were collected on a 3-Laser Cytek Northern Lights.

### 2.8. ELISA

Immulon 4 HBX plates (Thermo Scientific, Waltham, MA, USA) were coated with 50 µL/well of 1 µg/mL recombinant mCherry protein (Abcam Inc., Waltham, MA, USA, clone ab199750) at 4 °C overnight. Plates were washed with DPBS +0.05% Tween 20, before being blocked with DPBS +1% BSA (Sigma-Aldrich, Inc., St. Louis, MO, USA)) for 1 h at 37 °C. Serum was serially diluted by a factor of 2 beginning with a 1:50 dilution in DBPS +1% BSA. Diluted serum samples were incubated on blocked plates for 2 h at 37 °C. Plates were washed with DPBS +0.05% Tween 20, and anti-mouse IgG streptavidin-HRP-conjugated antibody (1:2000 in DBPS +1% BSA; Cell Signaling Technology) for 1 h at 37 °C. Plates were washed with DPBS +0.05% Tween 20, TMB substrate (BioLegend) was developed at 37 °C, stopped with 0.18 mol/L H_2_SO_4_, and read at 450 nm on an Agilent BioTek Cytation 7.

### 2.9. Immunohisotchemistry (IHC)

Endogenous peroxidases/phosphatases were quenched with BLOXALL blocking solution (Vector Laboratories, Inc., Newark, CA, USA), and tissues were blocked with Animal-Free Blocker R.T.U. (Vector Laboratories, Inc.). Sections were probed with primary antibodies against AR (D6F11, Cell Signaling Technology), CD8α (D4W2Z, Cell Signaling Technology), Foxp3 (FJK-16s, Invitrogen), or CD4 (D7D2Z, Cell Signaling Technology) overnight at 4 °C, washed with PBS, and incubated with the appropriate ImmPRESS polymer detection reagent (Vector Laboratories, Inc.) for 30 min at room temperature. Visualization was performed by incubation with 3,3′-diaminobenzidine (DAB) (Vector Laboratories, Inc.), ImmPACT Vector Red (Vector Laboratories, Inc.), or a Green HRP staining kit (Novus Biologicals, LLC, Centennial, CO, USA). For triple IHC, a second round of retrieval was performed with R-Buffer A after developing the first round of HRP and AP stains. Tissues were counterstained with Gill No. 3 Hematoxylin (Sigma-Aldrich), coverslipped and imaged on an Olympus IX73 inverted microscope or an Agilent BioTek Cytation 7 with a 20× objective. Infiltrated T cells were enumerated on 5 random fields of view per sample using ImageJ software (NIH, version 1.54k). Pixelwise H-Scores were calculated using QuPath (version 0.5.1) as previously described [18].

### 2.10. Quantitation and Statistical Analysis

Data were plotted and analyzed using GraphPad Prism v10. Details of statistical analyses can be found in the figure legends, and *p* values are displayed within the figures. *p* values of 0.05 or less were considered significant (* *p* < 0.05, ** *p* < 0.01, *** *p* < 0.001, **** *p* < 0.0001).

## 3. Results

### 3.1. Adenoviral Vaccines Targeting AR or AR-Vs Produce Antigen-Specific Immune Responses

**Figure 1:**
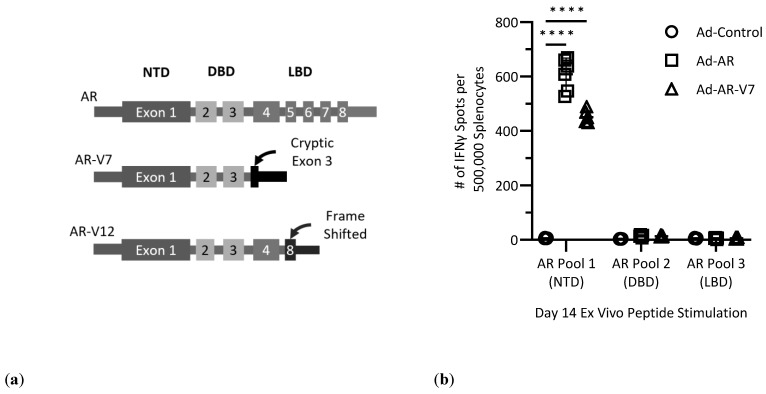

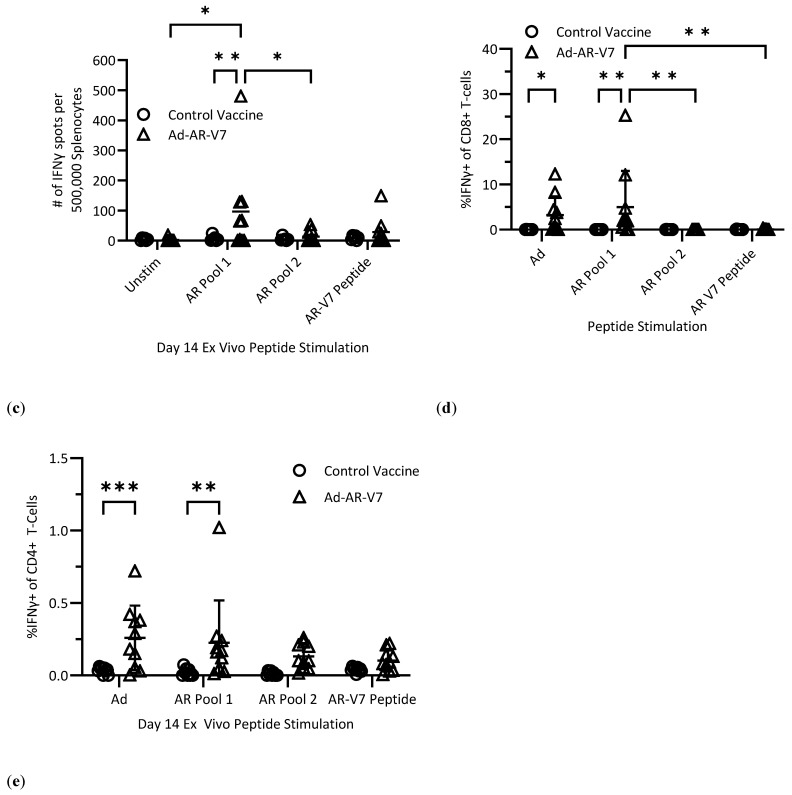
(**a**) Androgen receptor splice variant exon structures for AR, AR-V7 and AR-V12; (**b**) IFNγ ELISpot counts on splenocytes from B6 mice two weeks post-IM vaccination (*n* = 10); (**c**) IFNγ ELISpot counts on splenocytes from DO mice two weeks post-IM vaccination (*n* = 10, two-way ANOVA, with Bonferroni multiple comparisons); (**d**,**e**) Intracellular IFNγ+ staining of CD8+ T cells (**d**) or CD4+ T cells (**e**) from DO mice two weeks post-IM vaccination (*n* = 10, two-way ANOVA, with Bonferroni multiple comparisons) (* *p* < 0.05, ** *p* < 0.01, *** *p* < 0.001, **** *p* < 0.0001).

To explore the potential of AR and AR variants as vaccine targets and determine if tolerance against these proteins could be broken, we generated 1st-generation [E1,E3] adenoviral vectors encoding human AR, AR-V7, AR-V12 or a control antigens (Figure 1a). Using these vectors, we vaccinated naïve C57BL/6J mice intramuscularly and assessed antigen-specific T cell responses two weeks post-injection by IFNγ ELISpot using peptide pools corresponding to the N-terminal (NTD), DNA-binding/hinge (DBH) or ligand-binding domains (LBDs) of AR. These assays revealed that vaccination with Ad-AR, Ad-AR-V7 or Ad-AR-V12 all elicited significant responses to N-terminal domain peptides, although not against DNH or LBD peptides (Figure 1b). Additionally, we did not observe responses in this strain of mice to the cryptic epitope peptides of AR-V7, suggesting a strong immune dominance in C57BL/6J mice to N-terminal epitopes. To explore if epitope immune dominance was due to C57BL6 MHC-I restriction or background, we subsequently vaccinated JAX Diversity Outbred (DO) with Ad-AR-V7. Assessment of T cell responses from these mice included IFNγ ELISPOT as well as cytokine expression after in vitro stimulation, determined by intracellular flow cytometry. ELISPOT assays in DO mice revealed strong responses against Ad peptides (Figure S1), but with diverse individual responses against AR peptide pools (Figure 1c). While the majority of mice exhibited the most significant responses against N-terminal peptides, several mice exhibited responses against DBH peptides, with a larger number exhibiting responses against the AR-V7 cryptic epitope (Figure 1c). Flow-based assessments mirrored these responses and also indicated that IFNγ expression was due to both CD8+ and CD4+ T cell populations, although AR-V7 responses were specific for CD4+ T cells (Figure 1d,e). Notably, DO mice vaccinated with an irrelevant adenoviral vaccine did not exhibit significant immunity against AR peptide pools or an AR-V7 peptide (Figure 1c–e). Thus, these vaccines demonstrated the ability to elicit AR-specific and AR-V7-specific T cells responses in mice, which varied based on their genetic background and MHC-I repertoire, suggesting their potential utility against prostate cancer.

### 3.2. Preventative Vaccination with AR or AR-V7 Vaccines Can Elicit Protective Immunity Against AR-V7+ Cancers

**Figure 2:**
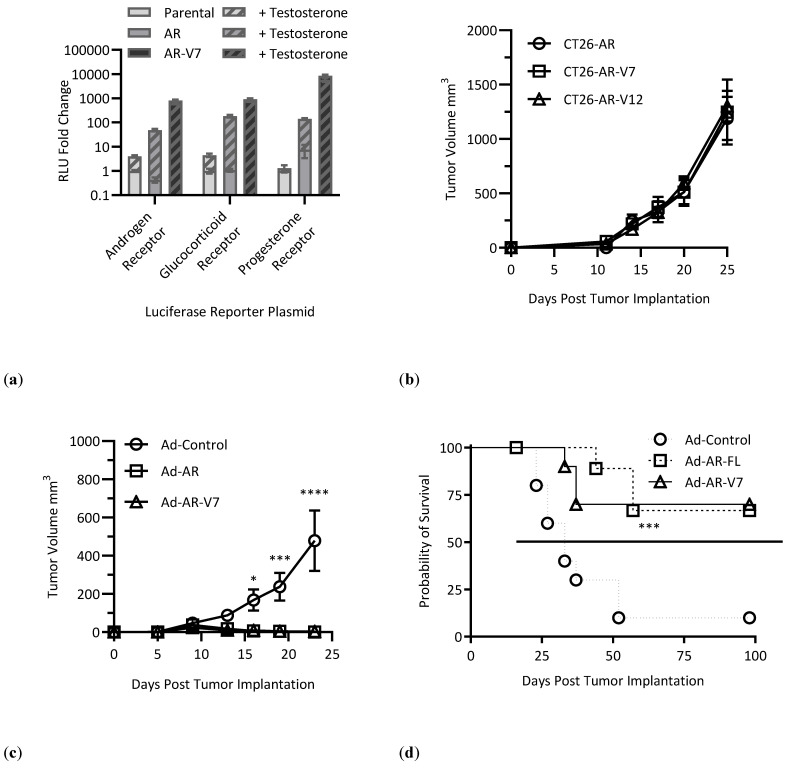

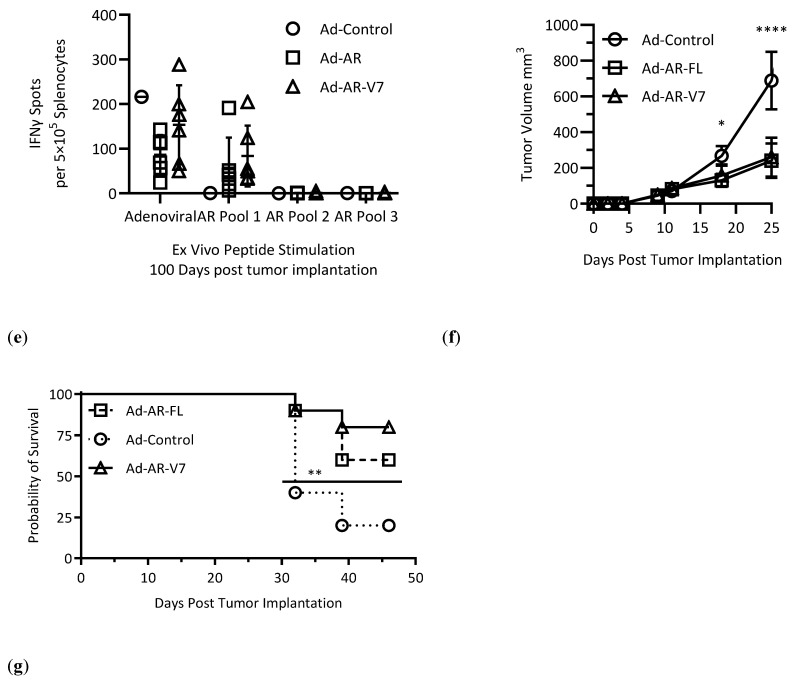
(**a**) Luciferase reporter assay on HEK293T cells shows that AR- and AR-V7-overexpressing lentiviral vectors result in strong signaling in ligand-dependent or ligand-independent manner, respectively; (**b**) Tumor growth curve of CT-26 tumors expressing AR, AR-V7 or AR-V12 in the flank of BALB/c male mice (*n* = 5); (**c**) CT-26-AR-V7 tumor growth curves in mice that were vaccinated IM two weeks prior to tumor implantation with Ad-AR, Ad-AR-V7 or Ad-Control (*n* = 10, two-way ANOVA, with Bonferroni multiple comparisons); (**d**) Kaplan–Meier plot of BALB/c male mice vaccinated before CT26-AR-V7 tumor implantation (*n* = 10, Gehan–Breslow–Wilcoxon test); (**e**) IFNγ ELISpot counts on splenocytes from tumor-rejecting mice 100 days post-tumor-implantation (*n* = 1, *n* = 6, *n* = 7 in Ad-Control, Ad-AR, and Ad-AR-V7, respectively); (**f**) CT26-AR-V7 tumor growth curves in mice that were vaccinated IM one day post-tumor-implantation with Ad-AR, Ad-AR-V7 or Ad-Control (two-way ANOVA, with Bonferroni multiple comparisons); (**g**) Kaplan–Meier plot of BALB/c male mice vaccinated after CT26-AR-V7 tumor implantation (*n* = 10, Gehan–Breslow–Wilcoxon test) (* *p* < 0.05, ** *p* < 0.01, *** *p* < 0.001, **** *p* < 0.0001).

To assess whether the AR-V7 antigen-specific responses we observed could produce anti-tumor responses, we first used a non-AR-expressing cell line (CT-26), which has been previously used to assess the efficacy of tumor-associated antigen immune responses [19]. We generated lentiviral vectors to stably transduce these lines to express AR, AR-V7, or AR-V12. Using these vectors, we first assessed a broad repertoire of 43 signaling pathways and identified that only in the absence of exogenous testosterone, both AR-V7 and AR-V12 conferred strong signaling for the AR, GR and PR pathways (Figure S2). Secondary studies using pathway reporters for these three dominant pathways revealed significant AR-specific signaling activity in the presence of testosterone for AR, but that only AR-V7 and AR-V12 showed significant signaling activity in the absence of testosterone (Figure 2a). Using these validated lentiviral vectors, we generated three stable CT-26 cell lines expressing AR, AR-V7 or AR-V12, and assessed their growth in vivo by implanting them subcutaneously in the flank of BALB/c male mice (Figure 2b). These in vivo studies revealed no difference in tumor growth due to AR expression or signaling; thus, we proceeded with vaccine studies using cell lines expressing AR-V7 as this is the most observed AR splice variant in mCRPC and produced the strongest induction of androgen signaling pathways (Figure S3).

As AR-V7 expression typically emerges in cases of advanced mCRPC after prolonged ADT, we first tested the prophylactic potential of our vaccine to prevent the growth of AR-V7+ cancer. For these studies, three groups of 10 BALB/c male mice were vaccinated IM with Ad-AR, Ad-AR-V7 or Ad-Control and CT-26-AR-V7 cells and subsequently implanted two weeks post-vaccination. These experiments revealed significantly inhibited tumor growth in both the Ad-AR- and Ad-AR-V7-vaccinated mice as compared to control vaccinated mice (Figure 2c), resulting in 8/10 and 7/10 mice remaining tumor-free from Ad-AR-and Ad-AR-V7-vaccinated mice, compared to 1/10 from control vaccinated mice (Figure 2d). Antigen-specific immune responses of these long-term survivors/tumor rejectors were assessed by IFNγ ELISpot, which again demonstrated immunodominant responses to the NTD peptide pool from BALB/c mice vaccinated with Ad-AR and Ad-AR-V7, as well as documenting the presence of long-term AR-specific immunity in vaccinated mice (Figure 2e).

Having observed the significant impact of prophylactic use of these vaccines, we next conducted a therapeutic vaccination study to simulate the impact of vaccination on patients with existing AR-V7+ cancers. In these studies, 30 mice were implanted with CT-26-AR-V7 cells and randomized to three groups of 10 that received IM vaccination with Ad-AR, Ad-AR-V7 or Ad-Control. These studies again revealed significantly inhibited tumor growth in both Ad-AR and Ad-AR-V7 treated groups compared to those receiving Ad-Control vaccines (Figure 2f), resulting in an overall median survival benefit for Ad-AR and Ad-AR-V7-vaccinated groups (Figure 2g). Collectively, these studies demonstrate that AR and AR-V7-specific vaccines are able to elicit antigen-specific immunity capable of significant therapeutic anti-tumor responses, even in models that do not rely upon androgen signaling.

### 3.3. Lentiviral Expression of AR-V7 in a Novel C57BL/6 Syngeneic Prostate Tumor Cell Line Provides an Immunosuppressive Benefit Compared to an Empty Lentiviral Control Cell Line

**Figure 3:**
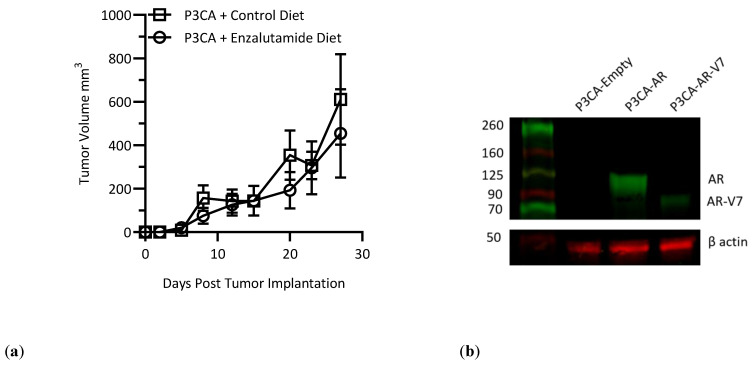

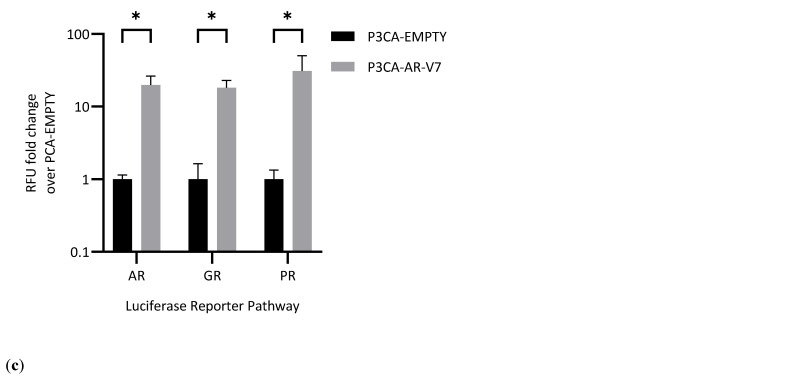

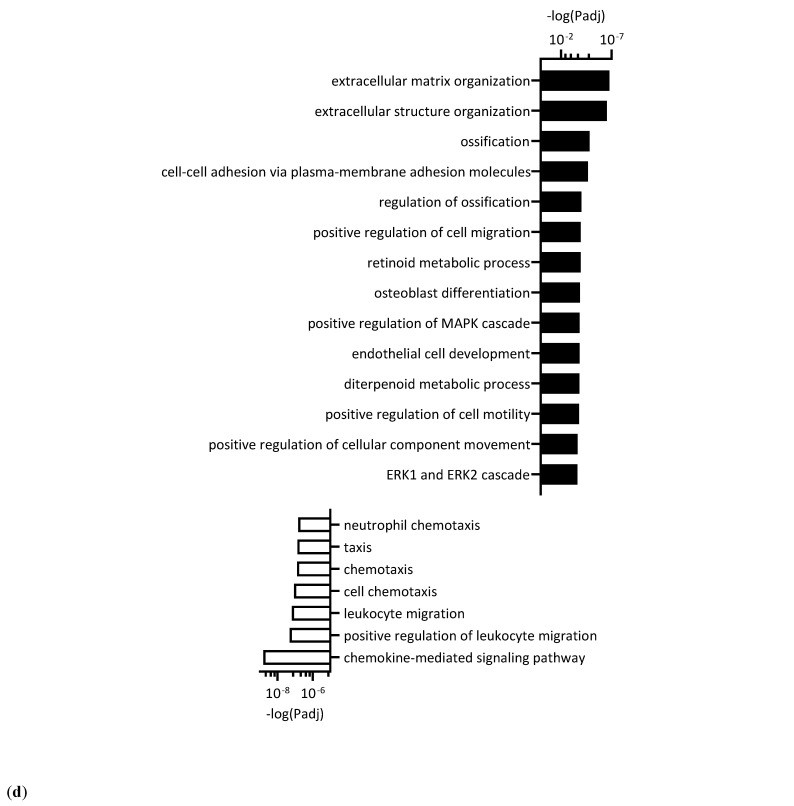

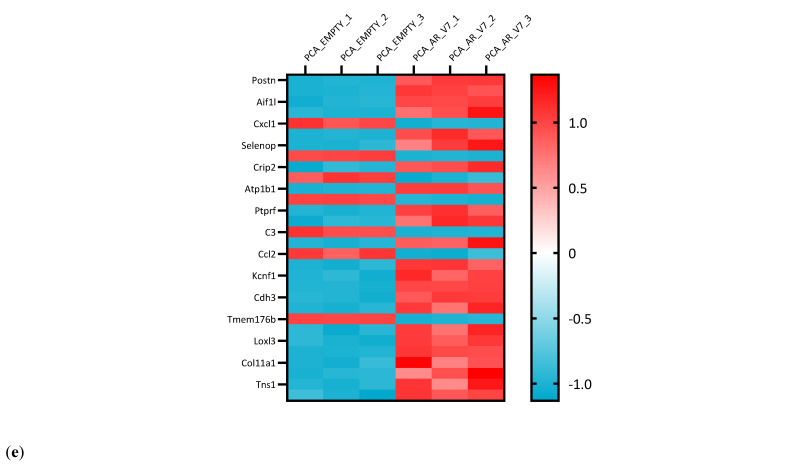
(**a**) Tumor growth curves for P3CA parental in B6 males with or without enzalutamide combination treatment (*n* = 5); (**b**) Western blot for AR in P3CA-Empty, P3CA-AR, and P3CA-AR-V7 cell lines; (**c**) Luciferase reporter assay on P3CA-Empty and P3CA-AR-V7 cell lines; (**d**) GO pathway analysis on differentially expressed genes up (black bars) and down (white bars) in P3CA-AR-V7 compared to P3CA-Empty; (**e**) Heatmap of the z-scores for the 30 most significantly differentially expressed genes between P3CA-Empty and P3CA-AR-V7 from bulk RNA sequencing (* *p* < 0.05).

To determine the impact of AR or AR-V7 in a prostate cancer model, we utilized our lentiviral vectors to express these genes in a murine cancer cell line, which was derived from a murine prostatic carcinoma. For these studies, we obtained a novel murine PTEN^−/−^ p53^−/−^ prostate cancer cell line (P3CA) developed by Dr. Ming Chen (Duke University) that was derived from a genetic mouse prostate cancer model syngeneic on C57BL/6. As expected from previous studies of PTEN KO prostate cancer models [20], we found that this cancer cell line was not AR-dependent, as it did not respond to enzalutamide in vivo when implanted subcutaneously in the flank of C57BL/6 males (Figure 3a). Using lentiviral vectors, three stable cell lines were derived from P3CA parental cells, P3CA-AR, P3CA-AR-V7 and P3CA-Empty, to express AR or AR-V7 or serve as an empty lentiviral control, respectively. We first confirmed expression of AR and AR-V7 by Western blot (Figure 3b), finding that AR-V7 was expressed, albeit at lower levels compared to AR. Given this reduction, we next assessed the induction of AR, GR and PR signaling pathways in AR-V7+ P3CA cells, in comparison to controls. These assays revealed that the presence of AR-V7, even at reduced expression levels, did allow for the significant stimulation of androgen-specific signaling pathways (Figure 3c). To assess the downstream impact of ligand-less androgen signaling, we performed next-generation RNA sequencing on P3CA-Empty and P3CA-AR-V7 cells, which demonstrated that AR-V7 significantly stimulated the expression of gene sets associated with extracellular matrix interactions, ERK signaling, cell adhesion, and cell motility, although with significant reduction in chemokine signaling and chemotaxis (Figure 3d), with suppression of different chemokine and inflammatory genes (such as CXCL1, CCL2, and C3), being represented in the 30 most significantly differentially expressed genes (Figure 3e). These alterations suggested potentially enhanced intrinsic growth and metastatic capacity, as well as a potential suppression of innate immunity within AR-V7-expressing prostate cancers.

**Figure 4:**
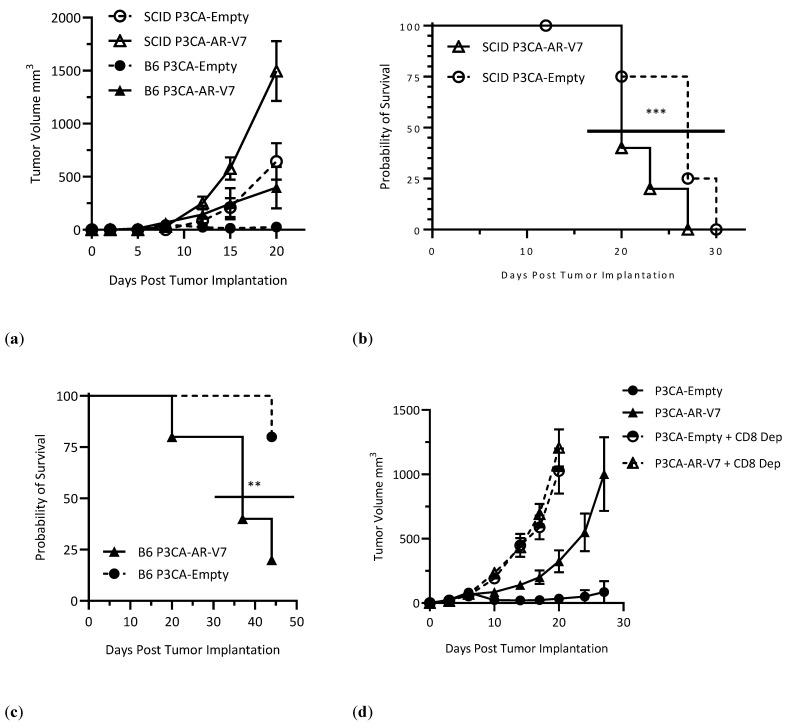

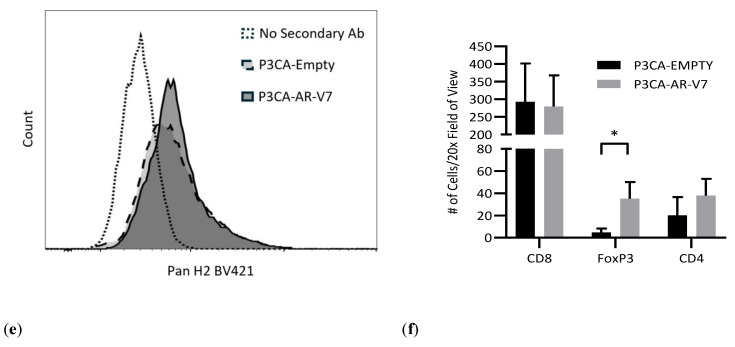
(**a**) Tumor growth curves for SCID and B6 albino male mice implanted subcutaneously with P3CA-Empty or P3CA-AR-V7 (*n* = 5); (**b**) Kaplan–Meier plot of SCID male mice with P3CA-Empty or P3CA-AR-V7 (*n* = 5, Log-rank test); (**c**) Kaplan–Meier plot of B6 albino male mice with P3CA-Empty or P3CA-AR-V7 (*n* = 5, Log-rank test); (**d**) Tumor growth curves for P3CA-Empty and P3CA-AR-V7 in B6 albino males with or without CD8 depletion (*n* = 5); (**e**) MHC-I expression on P3CA cell lines by flow cytometry (dotted—no secondary control, dashed—P3CA-Empty, solid—P3CA-AR-V7); (**f**) IHC quantification from P3CA-Empty and P3CA-AR-V7 tumors from B6 albino males, collected 7 days post-implantation (*n* = 5, unpaired t test with Welch correction and Holm-Šídák correction for multiple comparisons) (* *p* < 0.05, ** *p* < 0.01, *** *p* < 0.001).

To assess if these signaling alterations result in differences in tumor growth related to oncogenic capacity or immunity, we implanted P3CA-AR-V7 and P3CA-Empty cell lines into SCID and C57BL/6 mice. In the absence of adaptive immunity in SCID mice, we found that prostate AR-V7+ tumors grew significantly larger than control prostate tumors (Figure 4a). More critically, we found that while P3CA-AR-V7 cells implanted into immunocompetent C57BL/6J mice consistently formed tumors that continued to grow to humane endpoints, the majority of P3CA-Empty tumors began to grow but were eventually rejected (Figure 4c), likely due to the dual presence of mCherry and hygromycin resistance genes expressed in these cell lines, as we have documented with other xenoantigens [21]. As P3CA-AR-V7 cells also express these xenoantigens, these results suggest that AR-V7 expression provided some immunosuppressive benefit to allow P3CA-AR-V7 tumors to evade the adaptive responses that prevented P3CA-AR-Empty tumors from growing. Given the intracellular nature of mCherry and hygromycin, we hypothesized that this rejection could be due to the stimulation of CD8+ T cell responses. To assess if CD8+ cells mediated this rejection, we again implanted P3CA-AR-V7 or P3CA-Empty cells, in C57BL/6 males with or without the addition of an anti-CD8 antibody to deplete CD8+ T cells. Again, we observed that while P3CA-AR-V7 tumors grew, PC3A-Empty tumors were consistently rejected in non-depleted mice. Additionally, we found that CD8 depletion allowed for the equivalent development of both P3CA-Empty and P3CA-AR-V7 tumors (Figure 4d). Given a recent study suggesting an impact of AR on MHC-I expression [22], we first assessed if MHC-I suppression could account for the immune suppression of CD8+ T cells. Using flow cytometry, we were unable to detect significant differences in cell surface MHC-I expression between control and AR-V7-expressing P3CA cell lines (Figure 4e). Alternatively, our unbiased evaluation of RNAseq revealed alterations in multiple cytokines/chemokines (Figure 3d,e), suggesting that differences may involve AR-V7 suppression of T cell infiltration [23]. We collected tumor tissues from B6 albino males 7 days post-implantation, and stained for CD8, FoxP3, and CD4. We found that P3CA-AR-V7 tumors had significantly more FoxP3+ Tregs compared to P3CA-Empty tumors (Figure 4f and Figure S4). Given the immunosuppressive nature of this model, we next assessed if our vaccine could elicit anti-tumor efficacy in this stringent AR-V7+ P3CA model.

### 3.4. Androgen Receptor-Targeted Vaccination Combined with Enzalutamide or Anti-PD1 Checkpoint Inhibition

Although P3CA-AR-V7 tumors displayed an immunosuppressive phenotype enriched with FoxP3+ Tregs which allowed their growth in immunocompetent mice, we hypothesized that the antigen-specific responses produced by Ad-AR-V7 may be capable of eliciting anti-tumor responses in a preventative setting. To examine these effects, we vaccinated C57BL/6J male mice with Ad-AR, Ad-AR-V7 or Ad-Control and implanted mice with P3CA-AR-V7 cells 2 weeks post-vaccination. Notably, we found no difference in survival between Ad-AR-, Ad-AR-V7- or Ad-Control-vaccinated mice (Figure S5).

Given this lack of impact and studies suggesting that AR inhibition can prevent CD8 T cell exhaustion [24], we next tested the combination of Ad-AR or Ad-AR-V7 and enzalutamide. As in previous experiments with P3CA parental cells (Figure 3a), enzalutamide monotherapy did not show a significant impact on P3CA-AR-V7 tumor growth, nor did enzalutamide monotherapy enable P3CA-Empty tumors to avoid rejection (Figure S6).

**Figure 5:**
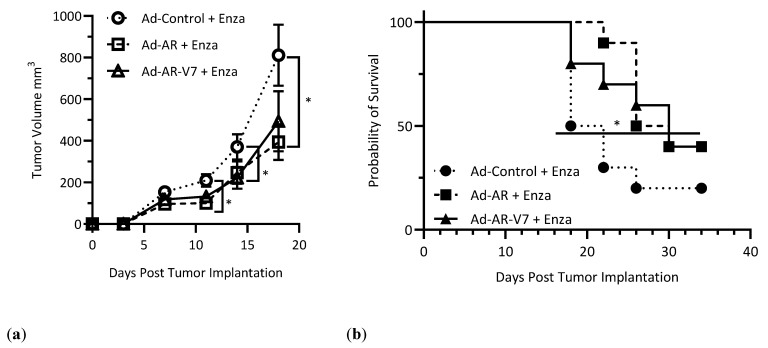

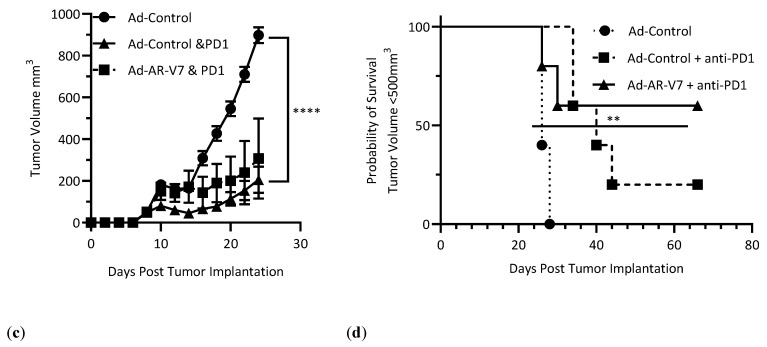
(**a**) Tumor growth curves of C57BL/6 male mice on enzalutamide-diet-vaccinated two weeks prior to P3CA-AR-V7 tumor implantation (*n* = 10, two-way ANOVA, with Bonferroni multiple comparisons); (**b**) Kaplan–Meier plot of C57BL/6 male mice on enzalutamide-diet-vaccinated two weeks prior to P3CA-AR-V7 tumor implantation (*n* = 10, Gehan–Breslow–Wilcoxon test); (**c**) Tumor growth curves of B6 albino male mice vaccinated two weeks prior to P3CA-AR-V7 tumor implantation, with or without anti-PD-1 antibody therapy (*n* = 5, two-way ANOVA, with Bonferroni multiple comparisons); (**d**) Kaplan–Meier plot of B6 albino male mice vaccinated two weeks prior to P3CA-AR-V7 tumor implantation, with or without anti-PD-1 antibody therapy (*n* = 5, Gehan–Breslow–Wilcoxon test) (* *p* < 0.05, ** *p* < 0.01, **** *p* < 0.0001).

However, we observed that vaccinated mice treated with enzalutamide demonstrated significantly smaller P3CA-AR-V7 tumors, and enhanced survival compared to controls (Figure 5a,b). Given the impact of this standard-of-care combination, we next evaluated the combination of Ad-AR-V7 with anti-PD1 checkpoint inhibition to enable CD8+ T cells in our P3CA-AR-V7 tumor model, as we have observed in other studies [15]. In these experiments, we found that anti-PD1 therapy could suppress the growth of P3CA-AR-V7 tumor growth compared to controls (Figure 5c), but that long-term survival was only altered in groups receiving combination Ad-AR-V7 and anti-PD1 treatment (Figure 5c,d). Notably, we observed complete tumor eradication in 60% of the mice in these groups, similar to heterogeneous responses observed in past preclinical and clinical studies utilizing anti-tumor vaccines and anti-PD1 therapies [15,25]. Consistent with our prior results, we observed that mice vaccinated with Ad-AR-V7 with the addition of anti-PD1 had significantly more AR-specific responses in IFNγ ELISpot assays on splenocytes collected at humane endpoints or 66 days post-tumor-implantation (Figure S7). Anti-mCherry serum IgG from mice at humane endpoint or at 66 days post-implantation was compared by ELISA. Although no statistically significant difference was observed between groups of tumor-bearing mice, serum from mice treated with Ad-Control and anti-PD1 or Ad-AR-V7 and anti-PD1 had significantly higher anti-mCherry IgG than tumor-naïve mice, demonstrating the induction of tumor-cell-specific adaptive immunity against these lines that was protected by AR-V7 expression (Figure S8). Tumors collected from these mice were stained for AR by IHC, and pixelwise H-scores were calculated. Although not statistically significant (*p* = 0.0605), Ad-Control and anti-PD1-treated tumors showed a trend toward lower pixelwise H-scores as compared with Ad-Control-treated tumors (Figure S9).

## 4. Discussion

In our studies, we determined that adenoviral vaccines targeting the androgen receptor (AR) and its splice variants (AR-Vs) could effectively elicit antigen-specific CD8 and CD4 T cell responses against AR epitopes and variant-specific epitopes for AR-V7. These responses were predominantly against the N-terminal domain epitopes of AR, but included responses against the AR-V7 cryptic epitope in outbred mice. This is in contrast to some previous clinical studies that have documented the existence and expansion of T cells against the AR-LBD epitopes in clinical samples [26]. As the androgen receptor is essential for the growth and development of most prostate cancers and plays a key role in other cancers, the induction of AR-directed immunity via vaccination may offer an effective means to immunologically stimulate responses against these cancers. While the induction of AR-specific immune responses may be more challenging in patients due to elevated immune tolerance in the tumor microenvironment, immunity against splice variant cryptic epitopes may be more easily achieved considering that protein expression of these epitopes is undetectable in benign tissues, which may signify a lower level of central immune tolerance against these epitopes. Tumor-restricted cryptic epitopes which are the result of aberrant RNA splicing may thus be more immunologically akin to neoantigens in cancer, representing compelling predictable targets for castration-resistant prostate cancer. Notably, the induction of responses against the N-terminus and AR-V7 cryptic epitope suggests the ability to target AR-V7. Vaccination targeting AR-V7 may be particularly impactful in mCRPC, where high expression of AR-V7 is common, and may offer a means to prevent the development of this type of resistance.

Using both preventative and therapeutic vaccination strategies, our studies revealed that Ad-AR or Ad-AR-V7 vaccination could elicit significant anti-tumor immune responses against AR-V7-expressing tumors. Notably, we found that these responses were most impactful in a preventative setting (Figure 2), suggesting that the establishment of local tumor immune suppression could strongly counteract induced T cell responses. In support of this finding, using an identical vaccination strategy, we found that anti-tumor immunity was less ineffective in an immune suppressive prostate cancer model. This suggests an elevated level of local CD8+ T cell exclusion in these cancers (Figure 4), which appears to be mediated by AR signaling (Figure 3). While our model is not dependent upon AR signaling for growth, our immunologic findings are congruent with clinical findings that have failed to demonstrate robust levels of infiltrating cytotoxic T cells in most prostate cancers [27], as well as a lack of clinical response to PD1 and PDL1 immune checkpoint inhibitors [28,29,30]. Currently, the role of androgen receptors in tumor immunity is unclear. While inhibition of AR signaling can lead to an immunosuppressive phenotype in myeloid cells [31], other studies have supported a role for AR conferring immunosuppression through the disruption of interferon signaling and suppression of MHC-I complexes [22,24]. Our studies suggest that androgen signaling may alter the innate immune microenvironment, although we did not observe a large shift in MHC-I expression (Figure 4e). Additionally, while we did not observe a strong effect from enzalutamide treatment, we did observe an effect of our vaccine in combination with enzalutamide. This demonstrates that the combination of the anti-androgen drug enzalutamide did not interfere with vaccine-induced anti-tumor immunity, highlighting the compatibility of these vaccines with standard CRPC treatments for resistant patients. Moreover, it suggests that AR-targeted vaccines could be integrated into existing therapeutic regimens, potentially enhancing overall treatment efficacy for prostate cancer patients in a metastatic setting, as well as after an early diagnosis. We also observed that anti-PD-1 immune checkpoint inhibition in combination with vaccination against AR-V7 resulted in complete tumor elimination in the majority of mice (Figure 5). Although immune checkpoint monotherapy has failed to show significant benefits in prostate-cancer-specific clinical trials [28,32,33], our studies suggest that vaccination against AR-V7 and potentially other AR variants can expand AR-specific T cells, which in combination with PD-1 immune checkpoint inhibition, allow for significant anti-tumor responses in AR-V7+ immunosuppressive tumor microenvironments. Vaccination likely activates and expands these AR-specific T cells, which likely remain naïve in the presence of immune checkpoint inhibitors. Our results in the preventative setting further suggest that once safety for these vaccines is established, it may be more effective to utilize these vaccines at earlier timepoints prior to the development of resistance, as a means to elicit AR-specific T cells against prostate cancer, as well as prevent the emergence of resistant AR-V7+ prostate cancer cells.

Overall, the experimental results suggest that adenoviral vaccines targeting AR and AR-Vs hold significant promise for the treatment of prostate cancer. These vaccines can induce robust, antigen-specific immune responses capable of preventing tumor growth and overcoming immunosuppressive mechanisms associated with AR-V7 expression. Furthermore, their compatibility with anti-androgen therapies positions them to be utilized in combination with current prostate cancer treatment strategies. As our results demonstrate that these vaccines are more effective in a preventative setting, it may be viable to utilize these vaccines early after prostate cancer diagnosis, in combination with standard-of-care treatment to immunologically stimulate responses as well as prevent the emergence of resistance, through the immune targeting of AR-V7 and other isoforms.

## 5. Conclusions

Using an adenoviral vaccine vector, we demonstrated that vaccines against AR and AR-V7 can elicit AR-specific T cell responses that can inhibit the growth of AR-V7-expressing tumors. Additionally, we showed that AR-V7 expression in a novel PTEN^−/−^ p53^−/−^ mouse prostate cancer cell line resulted in a more immunosuppressive tumor microenvironment with an increased number of FoxP3+ Tregs. Using this model, we demonstrated that vaccination in combination with anti-PD1 immune checkpoint inhibition resulted in the complete elimination of the majority of tumors. In conclusion, this work demonstrates the potential of vaccination against AR and AR-V7 as immunotherapies in prostate cancer and implies that vaccination against AR-V7 or other AR-Vs associated with treatment resistance could treat or inhibit the development of these resistance mechanisms, particularly in combination with immune checkpoint inhibitors.

## Data Availability

The raw data supporting the conclusions of this article will be made available by the authors on request.

## Supplementary Materials

The following supporting information can be downloaded at: https://www.mdpi.com/article/10.3390/vaccines12111273/s1, Figure S1: IFNγ ELISpot counts on splenocytes from DO mice two weeks post IM vaccination restimulated with adenovirus specific peptide or vehicle control (n = 5 & n = 9, Control Vaccine & Ad-AR-V7 respectively, 2 way ANOVA, with Bonferroni multiple comparisons); Figure S2: Luciferase reporter assay for all 45 pathways on 293T cell lines; Figure S3: Luciferase reporter assay on CT26-Empty and CT26-AR-V7 cell lines; Figure S4: Representative images of CD8 (brown), FoxP3 (red), and CD4 (green) IHC triple staining in (a) P3CA-Empty and (b) P3CA-AR-V7; Figure S5: Kapaln-Meier plot of C57BL/6J male mice, vaccinated with Ad-Control, Ad-AR or Ad-AR-V7, and implanted with PC3A-AR-V7 (*n* = 10, Log-rank test, ns); Figure S6: Tumor growth curves of C57BL/6 male mice implanted with P3CA-Empty or P3CA-AR-V7 with or without enzalutamide diet (*n* = 5); Figure S7: IFNγ ELISpot counts on splenocytes from B6 albino mice implanted with P3CA-AR-V7 vaccinated with Ad-Control or Ad-AR-V7 with or without anti-PD1, collected at humane endpoints or 66 days post implantation and restimulated with AR specific peptide pools (*n* = 5, 2way ANOVA, with Bonferroni multiple comparisons); Figure S8: ELISA for anti-mCherry serum IgG from B6 albino mice, implanted with P3CA-AR-V7, two weeks post vaccination, with or without anti-PD1 therapy. Control Serum collected from naïve mice. Serum was collected at humane endpoint, or 66 days post implantation; Figure S9: (a) IHC for AR in P3CA Tumors treated with Ad-Control, Ad-Control + anti-PD1, or Ad-AR-V7 + anti-PD1 (*n* = 5, *n* = 4, and *n* = 2, for Ad-Control, Ad-Control + anti-PD1, and Ad-AR-V7 + anti-PD1 respectively, ANOVA with Dunnett’s multiple comparisons); (b) Representative image for Ad-Control treated tumors; (c) Representative image for Ad-Control & anti-PD1 treated tumors; (d) Representative image for Ad-AR-V7 & anti-PD1 treated tumors.
