# Supplementary material for: Vaccination Against Androgen Receptor Splice Variants to Immunologically Target Prostate Cancer
Source: Vaccines (Basel). 2024 Nov 13;12(11):1273. doi: 10.3390/vaccines12111273 (PMC11599078; doi:10.3390/vaccines12111273)
Supplement: Supplementary file 1 [file vaccines-12-01273-s001.zip › Figures S1-S9.pdf]

vaccines

MDPI

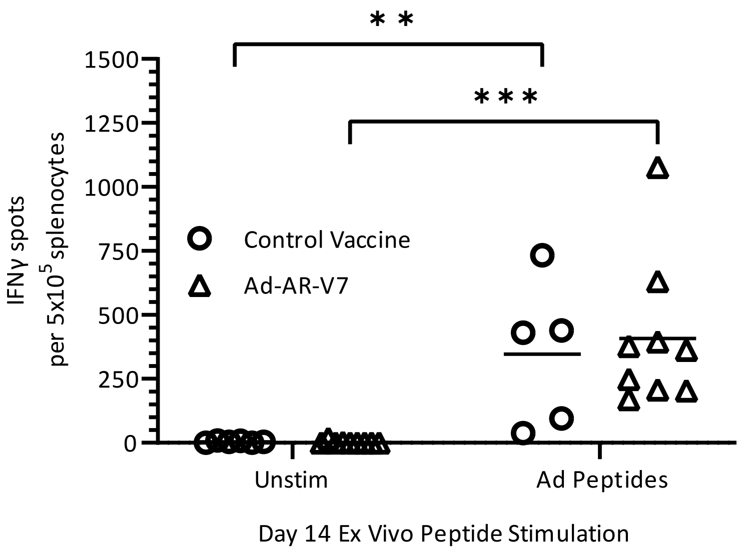

Figure S1. IFNγ ELISpot counts on splenocytes from DO mice two weeks post IM vaccination restimulated with adenovirus specific peptide or vehicle control (n = 5 & n = 9, Control Vaccine & Ad-AR-V7 respectively, 2 way ANOVA, with Bonferroni multiple comparisons)

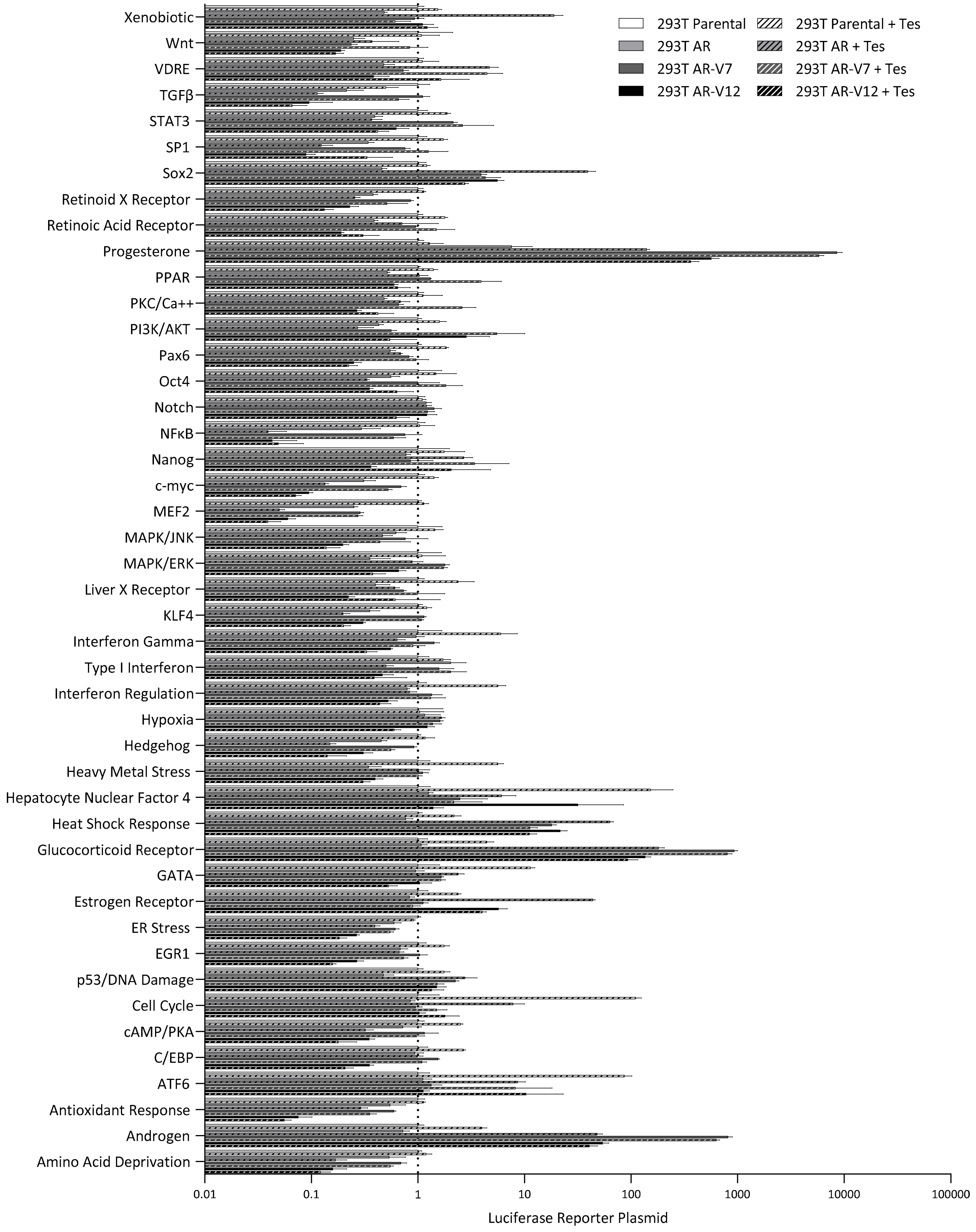

Figure S2. Luciferase reporter assay for all 45 pathways on 293T cell lines

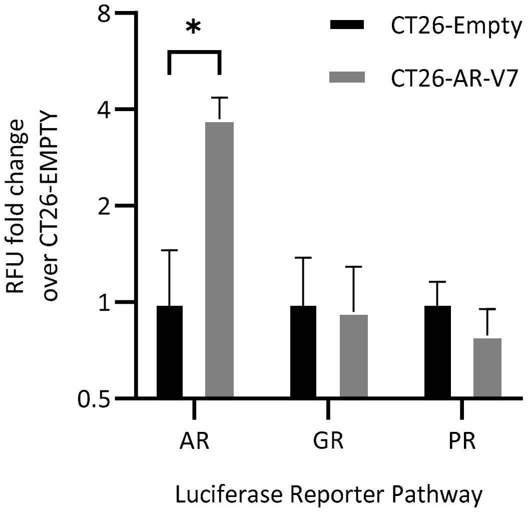

Figure S3. Luciferase reporter assay on CT26-Empty and CT26-AR-V7 cell linesAs AR-V7 expression typically emerges in cases of advanced mCRPC after prolonged ADT, we first tested the prophylactic potential of our vaccine to prevent the growth of AR-V7+ cancer. For these studies, three groups of 10 BALB/c male mice were vaccinated IM with Ad-AR, Ad-AR-V7 or Ad-Control and CT-26-AR-V7 cells and subsequently implanted two weeks post-vaccination. These experiments revealed significantly inhibited tumor growth in both the Ad-AR and Ad-AR-V7 vaccinated mice as compared to control vaccinated mice (Figure 2C), resulting in 8/10 and 7/10 mice remaining tumor free from Ad-AR and Ad-AR-V7 vaccinated mice, compared to 1/10 from control vaccinated mice (Figure 2D). Antigen-specific immune responses of these long-term survivors/tumor rejectors were assessed by IFNγ ELISpot, which again demonstrated immunodominant responses to the NTD peptide pool from BALB/c mice vaccinated with Ad-AR and Ad-AR-V7, as well as documenting the presence of long-term AR-specific immunity in vaccinated mice (Figure 2E).

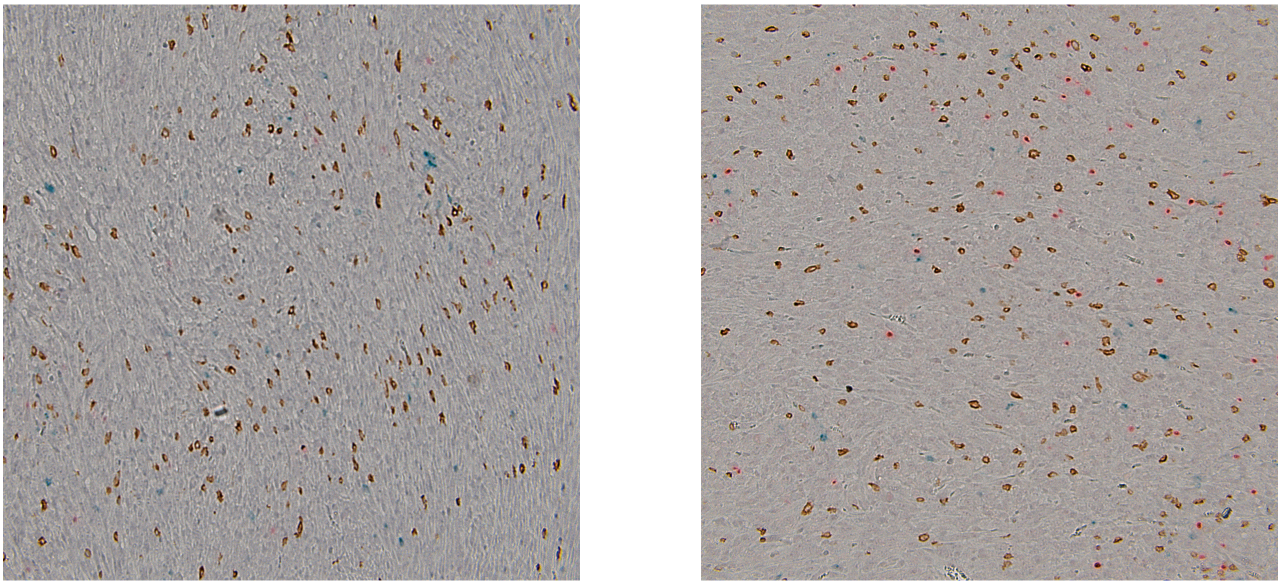

(a) (b)

Figure S4. Representative images of CD8 (brown), FoxP3 (red), and CD4 (green) IHC triple staining in (**a**) P3CA-Empty and (**b**) P3CA-AR-V7

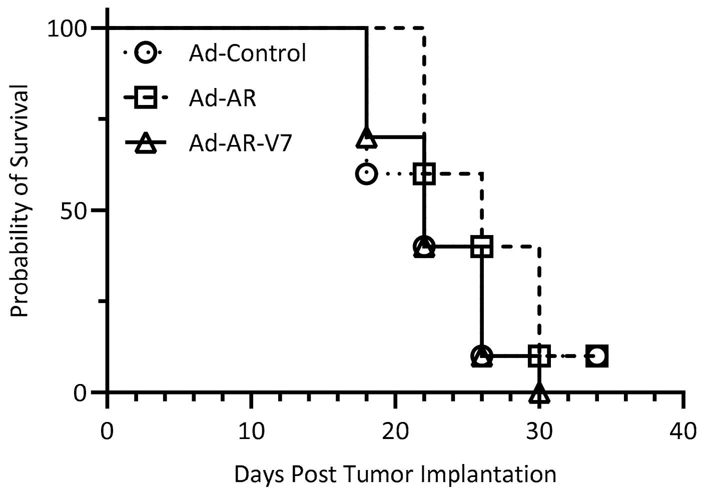

Figure S5. Kapaln-Meier plot of C57BL/6J male mice, vaccinated with Ad-Control, Ad-AR or Ad-AR-V7, and implanted with PC3A-AR-V7 (n=10, Log-rank test, ns)

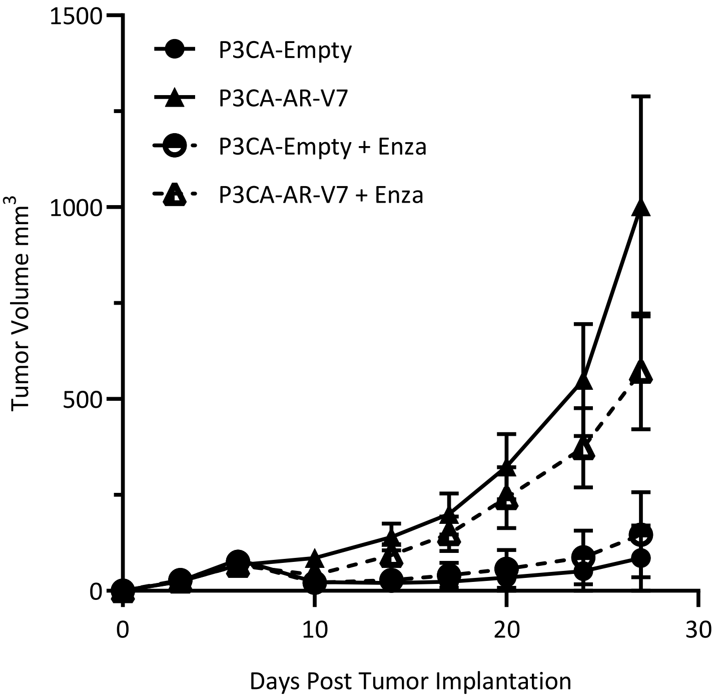

Figure S6. Tumor growth curves of C57BL/6 male mice implanted with P3CA-Empty or P3CA-AR-V7 with or without enzalutamide diet (n=5)

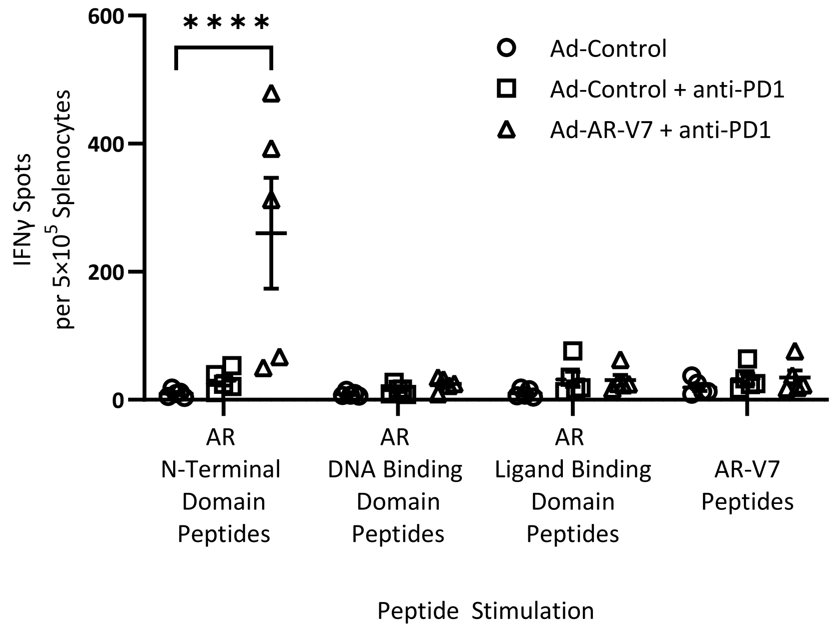

Figure S7. IFNγ ELISpot counts on splenocytes from B6 albino mice implanted with P3CA-AR-V7 vaccinated with Ad-Control or Ad-AR-V7 with or without anti-PD1, collected at humane endpoints or 66 days post implantation and restimulated with AR specific peptide pools (n=5, 2way ANOVA, with Bonferroni multiple comparisons)

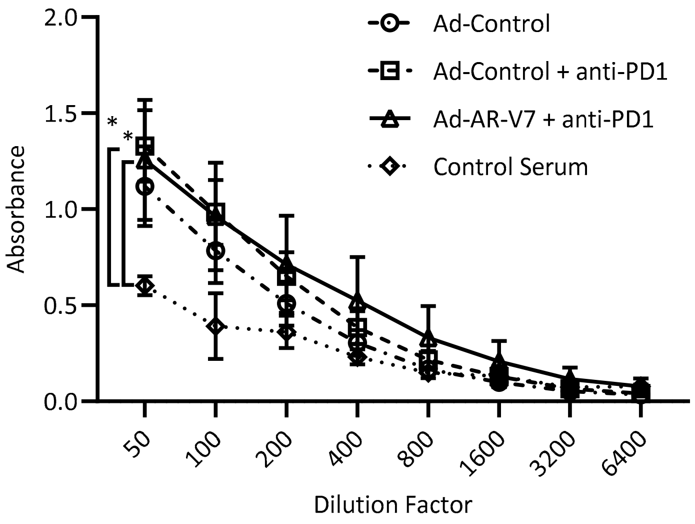

Figure S8. ELISA for anti-mCherry serum IgG from B6 albino mice, implanted with P3CA-AR-V7, two weeks post vaccination, with or without anti-PD1 therapy. Control Serum collected from naïve mice. Serum was collected at humane endpoint, or 66 days post implantation.

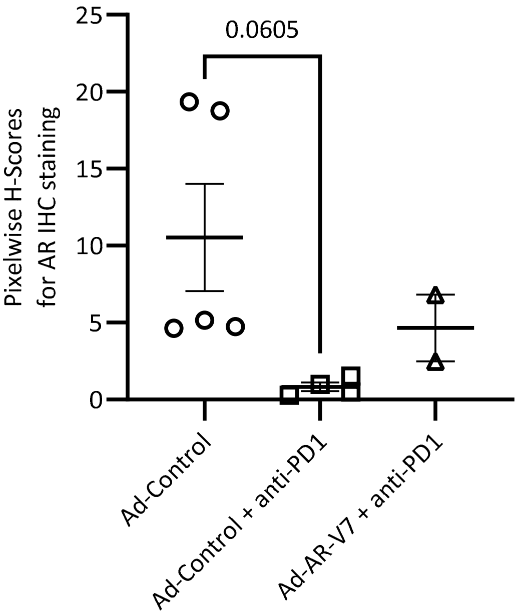

(a)

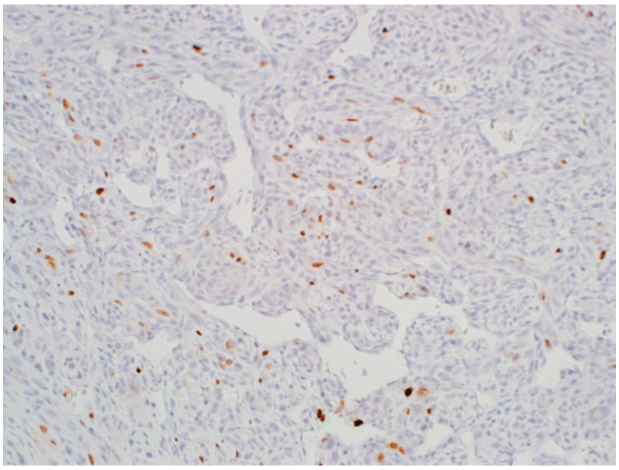

(b)

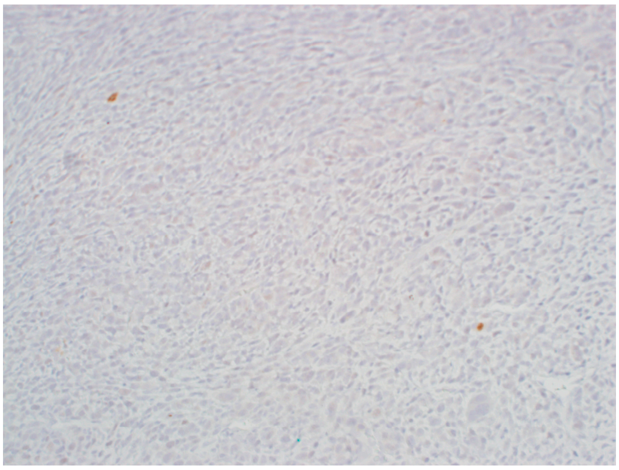

(c)

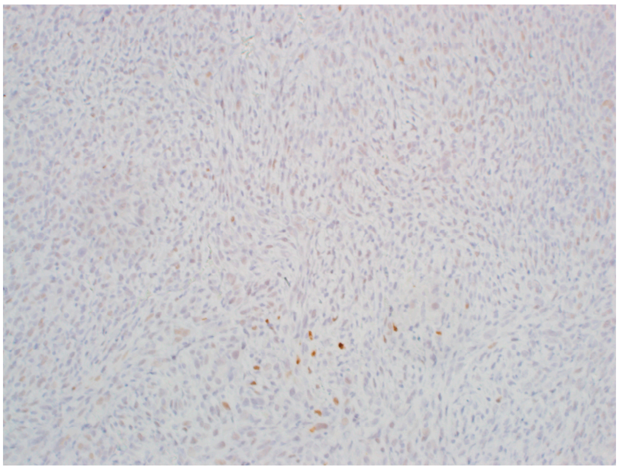

(d)

Figure S9. (**a**) IHC for AR in P3CA Tumors treated with Ad-Control, Ad-Control + anti-PD1, or Ad-AR-V7 + anti-PD1 (n=5, n=4, and n=2, for Ad-Control, Ad-Control + anti-PD1, and Ad-AR-V7 + anti-PD1 respectively, ANOVA with Dunnett's multiple comparisons); (**b**) Representative image for Ad-Control treated tumors; (**c**) Representative image for Ad-Control & anti-PD1 treated tumors; (**d**) Representative image for Ad-AR-V7 & anti-PD1 treated tumors

Disclaimer/Publisher's Note: The statements, opinions and data contained in all publications are solely those of the individual author(s) and contributor(s) and not of MDPI and/or the editor(s). MDPI and/or the editor(s) disclaim responsibility for any injury to people or property resulting from any ideas, methods, instructions or products referred to in the content.
