# Supplementary material for: Vaccination Against Androgen Receptor Splice Variants to Immunologically Target Prostate Cancer
Source: Vaccines (Basel). 2024 Nov 13;12(11):1273. doi: 10.3390/vaccines12111273 (PMC11599078; doi:10.3390/vaccines12111273)
Supplement: Supplementary file 1 [file vaccines-12-01273-s001.zip › Table S1.pdf]

Supplementary Table S1

Peptide Pool	AA Sequence
**AR Pool 1**	MEVQLGLGRVYPRPP
**AR Pool 1**	PRPPSKTYRGAFQNL
**AR Pool 1**	FQNLFQSVREVIQNP
**AR Pool 1**	IQNPGPRHPEAASAA
**AR Pool 1**	ASAAPPGASLLLLQQ
**AR Pool 1**	LLQQQQQQQQQQQQQ
**AR Pool 1**	QETSPRQQQQQQGED
**AR Pool 1**	QGEDGSPQAHRRGPT
**AR Pool 1**	RGPTGYLVLDEEQQP
**AR Pool 1**	EQQPSQPQSALECHP
**AR Pool 1**	ECHPERGCVPEPGAA
**AR Pool 1**	PGAAVAASKGLPQQL
**AR Pool 1**	PQQLPAPPDEDDSAA
**AR Pool 1**	DSAAPSTLSLLGPTF
**AR Pool 1**	GPTFPGLSSCSADLK
**AR Pool 1**	ADLKDILSEASTMQL
**AR Pool 1**	TMQLLQQQQQEAVSE
**AR Pool 1**	AVSEGSSSGRAREAS
**AR Pool 1**	REASGAPTSSKDNYL
**AR Pool 1**	DNYLGGTSTISDNAK
**AR Pool 1**	DNAKELCKAVSVSMG
**AR Pool 1**	VSMGLGVEALEHLSP
**AR Pool 1**	HLSPGEQLRGDCMYA
**AR Pool 1**	CMYAPLLGVPPAVRP
**AR Pool 1**	AVRPTPCAPLAECKG
**AR Pool 1**	ECKGSLLDDSAGKST
**AR Pool 1**	GKSTEDTAEYSPFKG
**AR Pool 1**	PFKGGYTKGLEGESL
**AR Pool 1**	GESLGCSGSAAAGSS
**AR Pool 1**	AGSSGTLELPSTLSL
**AR Pool 1**	TLSLYKSGALDEAAA
**AR Pool 1**	EAAAYQSRDYYNFPL
**AR Pool 1**	NFPLALAGPPPPPPP
**AR Pool 1**	PPPPPHPHARIKLEN
**AR Pool 1**	KLENPLDYGSAWAAA
**AR Pool 1**	WAAAAAQCRYGDLAS
**AR Pool 1**	DLASLHGAGAAGPGS
**AR Pool 1**	GPGSGSPSAAASSSW
**AR Pool 1**	SSSWHTLFTAEEGQL
**AR Pool 2**	EGQLYGPCGGGGGGG
**AR Pool 2**	GGGGGGGGGGGGGGG

**AR Pool 2**	GGGGGEAGAVAPYGY
**AR Pool 2**	PYGYTRPPQGLAGQE
**AR Pool 2**	AGQESDFTAPDVWYP
**AR Pool 2**	VWYPGGMVSRVPYPS
**AR Pool 2**	PYPSPTCVKSEMGPW
**AR Pool 2**	MGPWMDSYSGPYGDM
**AR Pool 2**	YGDMRLETARDHVLP
**AR Pool 2**	HVLPIDYYFPPQKTC
**AR Pool 2**	SGCHYGALTCGSCKV
**AR Pool 2**	SCKVFFKRAAEGKQK
**AR Pool 2**	GKQKYLCASRNDCTI
**AR Pool 2**	DCTIDKFRRKNCPSC
**AR Pool 2**	CPSCRLRKCYEAGMT
**AR Pool 2**	AGMTLGARKLKKLGN
**AR Pool 2**	KLGNLKLQEEGEASS
**AR Pool 2**	EASSTTSPTEETTQK
**AR Pool 2**	TTQKLTVSHIEGYEC
**AR Pool 3**	GYECQPIFLNVLEAI
**AR Pool 3**	LEAIEPGVVCAGHDN
**AR Pool 3**	GHDNNQPDSFAALLS
**AR Pool 3**	ALLSSLNELGERQLV
**AR Pool 3**	RQLVHVVKWAKALPG
**AR Pool 3**	ALPGFRNLHVDDQMA
**AR Pool 3**	DQMAVIQYSWMGLMV
**AR Pool 3**	GLMVFAMGWRSFTNV
**AR Pool 3**	FTNVNSRMLYFAPDL
**AR Pool 3**	APDLVFNEYRMHKSR
**AR Pool 3**	HKSRMYSQCVRMRHL
**AR Pool 3**	MRHLSQEFGWLQITP
**AR Pool 3**	QITPQEFLCMKALLL
**AR Pool 3**	ALLLFSIIPVDGLKN
**AR Pool 3**	GLKNQKFFDELRMNY
**AR Pool 3**	RMNYIKELDRIIACK
**AR Pool 3**	IACKRKNPTSCSRRF
**AR Pool 3**	SRRFYQLTKLLDSVQ
**AR Pool 3**	DSVQPIARELHQFTF
**AR Pool 3**	QFTFDLLIKSHMVSV
**AR Pool 3**	MVSVDFPEMMAEIIS
**AR Pool 3**	EIISVQVPKILSGKV
**AR Pool 3**	SGKVKPIYFHTQ
**AR-V7 Peptide**	YEAGMTLGEKFRVGNCKHLKMTRP
